# Flavonoids: Potential Candidates for the Treatment of Neurodegenerative Disorders

**DOI:** 10.3390/biomedicines9020099

**Published:** 2021-01-20

**Authors:** Shweta Devi, Vijay Kumar, Sandeep Kumar Singh, Ashish Kant Dubey, Jong-Joo Kim

**Affiliations:** 1 Systems Toxicology and Health Risk Assessment Group, CSIR-Indian Institute of Toxicology Research, Lucknow 226001, India; sweta.kamal123@gmail.com; 2 Department of Biotechnology, Yeungnam University, Gyeongsan, Gyeongbuk 38541, Korea; 3 Department of Medical Genetics, SGPGIMS, Lucknow 226014, India; sandeepcbt@gmail.com; 4 Department of Neurology, SGPGIMS, Lucknow 226014, India; ashish.icom@gmail.com

**Keywords:** flavonoids, cellular stress response, neurodegenerative disorders, ER stress proteotoxicity, oxidative stress, neuroinflammation

## Abstract

Neurodegenerative disorders, such as Parkinson’s disease (PD), Alzheimer’s disease (AD), Amyotrophic lateral sclerosis (ALS), and Huntington’s disease (HD), are the most concerning disorders due to the lack of effective therapy and dramatic rise in affected cases. Although these disorders have diverse clinical manifestations, they all share a common cellular stress response. These cellular stress responses including neuroinflammation, oxidative stress, proteotoxicity, and endoplasmic reticulum (ER)-stress, which combats with stress conditions. Environmental stress/toxicity weakened the cellular stress response which results in cell damage. Small molecules, such as flavonoids, could reduce cellular stress and have gained much attention in recent years. Evidence has shown the potential use of flavonoids in several ways, such as antioxidants, anti-inflammatory, and anti-apoptotic, yet their mechanism is still elusive. This review provides an insight into the potential role of flavonoids against cellular stress response that prevent the pathogenesis of neurodegenerative disorders.

## 1. Introduction

Neurodegenerative disorders are marked by different clinical features including memory and cognitive impairment, motor dysfunction, speaking disability, and breathing problems [1,2,3,4,5]. These symptoms are the consecutive results of stress conditions. Exposure to any stress, such as oxidative stress, environmental stress (metals and pesticides), and pharma chemicals, lead to disruption of cellular homeostasis by changing the normal cellular function. Cellular homeostasis includes neuroinflammation, protein quality control (PQC), and endoplasmic reticulum (ER) stress that are consistent in combating stress conditions [6,7,8,9,10]. A compromised cellular stress response condition leads to an imbalance in cellular homeostasis that results in cell death. Recent studies have found that flavonoids can prevent cell death by attenuating the cellular stress response [11,12]. Natural flavonoids are present in food and these are the most ingested polyphenolic compounds. These flavonoids have many therapeutic properties, such as anti-microbial, anti-oxidant, anti-inflammatory, and immune-modulatory [13,14,15,16,17,18,19]. Recent studies show the effectiveness of flavonoids in neurodegenerative disorders [20]. Diet rich in flavonoids have shown benefits against oxidative stress, inflammation [21], cardiovascular disease [22,23,24], apoptosis, and cancer [25,26]. The potential roles of flavonoids in neurodegenerative disorders were also confirmed by many studies. Citrus flavonoids, such as naringenin and hesperidin, both can cross the blood-brain barrier (BBB) and prevent neuronal deterioration [15,27,28,29]. Nobiletin (citrus flavonoid) shows the anti-neuroinflammatory effect by alleviating the inflammatory response. These pieces of evidence suggest that the therapeutic property of flavonoids against cellular stress and that could be used as a targeted drug for neurodegenerative disorders. This review provides insight on those flavonoids that prevent cellular death by alleviating the toxic impact of the cellular stress response.

## 2. Search Strategy

A comprehensive literature search was conducted to identify relevant research articles showing the beneficial effects of flavonoids in different models of neurodegenerative diseases. We searched Web of Science, PubMed, Google Scholar, Embase, and Cochrane Library databases to identify all relevant studies. We used different keywords for the search, such as “neurodegeneration, neuroprotective, neuroprotection, and neurodegenerative diseases, combined with “bioflavonoids, flavonols, flavan-3-ols, anthocyanin, flavone, flavones, isoflavones isoflavonoids or flavonones, and flavonoid”. Studies were included by studying the abstracts of the collected articles.

### Selection Criteria

All studies showing the effects of flavonoids on in vitro and in vivo models of neurodegenerative disease were selected. Administration of drugs, mode of administration, and treatment schedule were not considered. Studies conducted on any species, age, and sex were included. Studies where a comparison between different groups was given (e.g., control group, diseased group, and treated with flavonoids group) were included. We did not include incomplete data, unpublished data, abstracts, conference proceedings, commentary, editorials/letters, and duplicate references.

## 3. Environmental Stress and Cellular Stress Response

### 3.1. Environmental Stress

A gradual rise in hazardous chemicals, such as heavy metals, pesticides, and pharma chemicals, causes an imbalance in the environment that adversely affects human health [30]. Epidemiological studies have suggested that environmental chemical exposure to humans was associated with several disorders. The toxic effect of these chemicals is due to the imbalance in cellular stress response. Cells have a well-evolved cellular homeostasis system; however, stress exposure leads to disruption of cellular homeostasis by causing an imbalance between the reactive oxygen species (ROS) and the antioxidant system. Under oxidative stress, the generation of superoxide radical (•O2^−^) in mitochondria is the former step in the formation and proliferation of other ROS. These free radicals react with hydrogen peroxide (H_2_O_2_) via the iron-catalyzed Haber–Weiss reaction that generates the hydroxyl radical (•OH) [31,32]. Another ROS ‘peroxynitrite’ (ONOO^−^) formation is accompanied by the reaction of free radical nitric oxide and O_2_. The presence of peroxynitrite causes severe toxic effects due to its interactions with amino acids that alter the structure/function of the protein [33,34]. Exposure to pesticides and heavy metals leads to a rise in ROS production. These ROS cause irreversible damage to the cellular macro-molecules that are associated with the alteration of mitochondrial membrane functions, thus causing mitochondrial dysfunction and apoptosis [35,36,37]. These environmental stress factors also induce proteotoxicity by altering the structure of proteins, or affecting the nascent polypeptide chain folding, e.g., arsenic induces protein aggregation [38]. Furthermore, studies show that arsenic exposure causes protein misfolding that might affect protein-protein interactions, thereby causing proteotoxicity and thus affecting cell viability [39,40]. Cadmium exposure to yeast cells leads to the unfolded protein response induction through impairment of protein folding in the endoplasmic reticulum [41,42]. Exposure to chromium results in protein damage by oxidation. Chromium also induces protein aggregation by enhancing mRNA mistranslation. Mistranslation appears to be a primary cause of cellular chromium toxicity [43]. Copper toxicity induces oxidative stress, inflammation, apoptosis, astrocytosis, and excitotoxicity in the corpus striatum, hippocampus, and frontal cortex region of the brain [44,45]. Pesticides are also known to show similar effects. Rotenone and dieldrin induce the aggregation of alpha-synuclein and mutant huntingtin (mthtt) protein [46,47]. Paraquet treatment of SHSY-5Y cells induces the decrease in levels of proteasome 19S subunits and causes proteasome dysfunction [48]. Thus, exposure to environmental or intracellular stress could initiate the cellular stress response to protect the cellular homeostasis, while exaggerated stress conditions could lead to cell death.

### 3.2. Cellular Stress Response

Cells eliminate toxic substances in many ways. Several types of stress, such as heat stress, provoke various protective responses including oxidative stress response, heat shock response, and unfolded protein response (UPR). All these stress responses work to balance the cellular homeostasis either by monitoring and protecting the protein quality control or by neutralizing the toxic effect of reactive nitrogen and oxygen species (RNS; ROS). These heat shock responses and UPR are generally enhanced either by intercellular (oxidative stress) or, extra-cellular (pesticide/metals) stresses. Both stress conditions lead to disruption of the PQC by damaging the protein and making large aggregates. Under stress conditions, cells enhance the expression of various heat shock proteins (HSPs) that maintain the protein structure and refolds a misfolded protein. These HSPs are grouped into different subfamily according to their molecular weight. These include: HSP110, HSP90, HSP70, HSP60, HSP40, and small HSPs (sHSPs). All these HSPs are ATP-dependent except the sHSPs [49,50,51,52]. Hsp27, Hsp70, and Hsp32 (Heme Oxygenase, HO-1) are generally responding to neuronal injuries including ischemia and hemorrhage. Hsp27 is a sHSP and works by making a multimer post phosphorylation [53]. HSP90 is associated with the maturation of substrates, especially those that have a role in various cellular pathways, such as E3 ubiquitin ligases, kinases, and transcription factors. HSP90 attains certain specific conformational states that are stabilized by co-chaperones [54,55].

Exposure to stress that interferes with the glucose level, protein glycosylation, and Ca^2+^ disturbance causes the accumulation of unfolded proteins in the endoplasmic reticulum. This results in the activation of the UPR [56]. This UPR activates a set of different proteins including inositol-requiring protein-1 (IRE1), protein kinase RNA (PKR)-like ER kinase (PERK), and activating transcription factor 6 (ATF6). UPR signaling protects a cell from an imbalanced unfolded protein load by increasing the folding capacity of the ER [57,58]. However, excessive protein overload in the ER or defects in the UPR may induce cell death, known as ER stress-induced cell death.

Generally, cells maintain a healthy balance by monitoring the ratio of pro-oxidant: antioxidant levels, but oxidative stress arises when the cells’ antioxidant systems, such as superoxide dismutase (SOD), glutathione peroxidase (GPx), catalase, and other antioxidant proteins, fail to work [59]. ROS and RNS may interfere with the electron transport system. Furthermore, ROS and RNS also induce peroxidation of lipids in the plasma membrane and impair the functional activities of DNA and proteins [30,60,61]. All these cellular stress responses try to protect the cell from stress, but under extreme conditions, the cellular defense system fails to recover, thus promoting cell death.

## 4. Flavonoids

Flavonoids are polyphenolic compounds present in plants and are synthesized by the phenylpropanoid pathway [62,63,64]. They have antioxidative and anti-inflammatory properties [63,64,65]. Several case studies suggest that the intake of flavonoids reduce the risk of dementia [66]. Flavonoids have a neuroprotective property and they reduce the oxidative stress in epilepsy. In the central nervous system (CNS) several flavonoids bind to the benzodiazepine site on the γ-Aminobutyric acid type A (GABAA)-receptor resulting in anticonvulsive effects [67]. Intake of berry flavonoids improves memory in elderly people. Dietary cocoa flavanols improve cognition in older adults by enhancing dentate gyrus function [68]. Intake of cocoa flavanols improves human cognition and counteracts different types of cognitive decline [69]. Gratton et al. found that intake of cocoa flavanols enhances cerebral cortical oxygenation and cognition in healthy adults [70].

Flavonoids are categorized into different subgroups, summarized in Table 1. The application of flavonoids could mitigate the harsh effect of stress-induced cellular events. Hence, the use of these flavonoids could attenuate the toxic effect of environmental stress and cellular stress response.

## 5. Flavonoids and Cellular Stress Response

### 5.1. Role of Flavonoids in Neuroinflammation

Neuroinflammation is an immune response of the CNS. During neuroinflammation, glial cells (microglia) get activated and release inflammatory mediators, such as cytokines, chemokines, and ROS/RNS [86]. The flavonoids can interact with neuronal receptors and modulate kinase signaling pathways, transcription factors, and gene and/or protein expression, which control memory and learning processes in the hippocampus [87]. The level of prostaglandins (PGs) increases in the inflamed neuronal region, a feature of acute inflammation [88]. In an aging brain, neuroinflammation is marked by an increase in prostaglandin E2 (PGE2) levels. Once the neuroinflammation achieves the threshold and becomes over-activated, it leads to cellular damage and loss of neuronal function. Microglia activation/proliferation and reactive astrogliosis are commonly observed during neuroinflammation. Activated microglia are involved in the onset and maintenance of astrocyte proliferation. Lipopolysaccharide treatment in primary enriched astrocyte cultures results in increased proliferation of astrocytes. PGE2 released from activated microglia enhances astrocyte proliferation [89].

Flavonoids have a neuroprotective role in both in-vitro and in-vivo models against neuroinflammation [15,65,90]. Flavonoids can suppress the microglial activation and reduce the neurotoxicity induced by neurotoxic species released by microglia. The plant flavonoid wogonin inhibits activation-induced death of C6 glial cells by suppressing nitric oxide (NO) production. These inhibitory effects of wogonin on NO production are exerted through inhibition of NF-kappaB-mediated inducible nitric oxide synthase (iNOS) induction [91].

Flavonoids, luteolin, and apigenin protect the dopaminergic neurons by reducing oxidative stress, neuroinflammation and microglial activation along with enhanced neurotrophic potential in MPTP (1-methyl-4-phenyl-1,2,3,6-tetrahydropyridine) induced parkinsonism mice model. Luteolin and apigenin-treated mice model shows increased brain-derived neurotrophic factor (BDNF) levels in the substantia nigra region of the brain compared to MPTP treatment mice [92]. Li et al. found that treatment with apigenin (20 mg/kg, intragastrically) for three weeks remarkably ameliorated chronic unpredictable mild stress (CUMS)-induced behavioral abnormalities, such as: decreased locomotor activity and reduced sucrose consumption. Apigenin inhibits IL-1β and caspase-1 via disrupting the NLRP3 assembly. Apigenin inhibits the NLRP3 (NOD-, LRR-, and pyrin domain-containing protein 3) inflammasome activation through the upregulation of peroxisome proliferator-activated receptor-gamma (PPARγ) [93]. Apigenin ameliorated dopaminergic neuronal loss and improved behavioral, biochemical, and mitochondrial enzyme activities by suppression of oxidative stress and neuroinflammation [94].

Another flavonoid, rutin, when given to male albino Wistar rats, decreases mRNA expression of cytokines, caspase-1, apoptosis-associated speck-like CARD-containing protein (ASC), and ASC-NLRP3 [95]. Daidzein (flavonoid) ameliorates the inflammatory process and alleviates the risk of Alzheimer’s disease (AD) progression. Daidzein treatment down-regulates the expression of TNF-α, IL-1, and IL-6 in the primary astrocytes which are stimulated with amyloid-beta or lipopolysaccharide [96]. Catechin (flavonoid) protected murine microglia N9 cells from tert-butylhydroperoxide induced cell death by the inhibition of NF-kB, p53 activity, and activation of extracellular signal-regulated protein kinase (ERK) [97]. Blueberry extract (rich in flavonoids) inhibits the production of inflammatory mediators iNOS and COX-2 and reduces the level of NO, TNF-α, IL-1β, and ROS in lipopolysaccharide-activated BV2 microglial cells [98].

Naringenin treatment prevents neuronal cell death in LPS/IFNγ stimulated glial cells by the reduction in iNOS, NO, and TNF-α level and inhibition of p38 signaling cascades and STAT-1 transcription factor [99]. Biochanin A protects dopaminergic neurons against LPS-induced damage through inhibition of microglia activation and reduction in superoxide, TNFα, and NO [100]. Nobiletin prevents neuroinflammation in LPS-stimulated BV-2 microglial cells by inhibiting the release of TNF-α, IL-1β, ERK, c-Jun NH(2)-terminal kinase (JNK), and p38 mitogen-activated protein kinases (MAPKs) [101]. Adjunctive treatment with genistein and daidzein preserve neuronal functioning and sustain neurocognitive abilities of HIV-1 infected persons via a selective ER-mediated mechanism in neurons [102].

Transgenic Parkinson’s disease (PD) mice (C57BL/6 mice) received grape polyphenol concentrate (1.5 mL/kg/day) from the age of 6–8 weeks for four months have improved their behavioral and cognitive function. Grape polyphenol exhibits neuroprotective activity by reducing the α-synuclein accumulation in the frontal cortex and neuroinflammatory response in the frontal cortex and hippocampus [103]. Luteolin protects dopaminergic neurons against inflammation-induced neurotoxicity by inhibiting microglial activation [104]. Naringin (present in grape and orange) protects dopaminergic neurons by induction of the activation of the mammalian target of rapamycin complex-1 and inhibited microglial activation in the 6-OHDA treated mouse model [105]. Naringenin protects against 6-OHDA-induced neurotoxicity via activation of the nuclear factor E2-related factor 2 (Nrf2) and antioxidant response element (ARE) signaling pathway [106]. Baicalein inhibits the upregulation of tumor necrosis factor-α and interleukin-1β in the substantia nigra and striatum in MPTP-induced PD mice models [107]. Baicalein inhibits α-synuclein aggregation, inflammasome activation, and cathepsin B production in Sprague-Dawley rats treated with 1-methyl-4-phenylpyridinium [108].

### 5.2. Role of Flavonoids in Oxidative Stress

ROS are the major cause of oxidative stress and are linked with the pathogenesis of several neurological disorders [109]. Accumulation of ROS, such as hydroxyl radicals (•OH), superoxide radicals (•O2−), and hydrogen peroxide (H_2_O_2_), are associate with neuronal cell death [110,111]. The elevation in ROS induces protein oxidation, DNA damage, and lipid peroxidation (LPO), collectively leading to apoptosis in neuronal cells [112]. Uses of antioxidants, such as flavonoids, might be beneficial in reducing the toxicity of the oxygen-free radicals. These flavonoids have the potential to counter the toxicity of oxidative stress and decrease the pathogenesis of several neurological disorders [113]. Treatment with flavonoids, namely quercitrin, isoquercitrin, and afzelin, in human neuronal SH-SY5Y neuronal cells has shown beneficial effects through regulating inflammation, apoptosis, and ROS-scavenging. These flavonoids attenuated inflammation by inhibiting the expression of nitric oxide synthase, cyclooxygenase-2, and caspase activation [114]. Treatment with quercetin and luteolin and their metabolites 3,4-dihydroxytoluene (DHT) and 3,4- 3,4-dihydroxyphenylacetic acid (DHPAA), respectively, in neuronal PC12 cells, prevents oxidative stress. These metabolites are less efficient than parent flavonoids [115].

Two novel prenylated flavonoids, morachalcone D and morachalcone E, isolated from mulberry leaf, have antioxidant properties since their exposure to HT22 cells. Morachalcone D has higher efficiency than morachalcone E as it inhibits glutamate and erastin-induced cellular damage. Morachalcone D inhibits ROS production, glutathione (GSH) depletion, and iron accumulation. It is also involved in the upregulation of the expression of several genes of the antioxidant systems including *Nrf2, GPx4, SOD2, SLC7A11, HMOX1,* and *CAT* [116].

Phloretin and phlorizin (dihydrochalcone, a type of natural phenol, a dihydrochalcone, a family of bicyclic flavonoids) have neuroprotective effects against rotenone-induced toxicity in human SH-SY5Y neuroblastoma cells. They reduce rotenone-induced cell death by actively scavenging ROS, normalizing mitochondrial transmembrane potential, inhibiting caspase 3 activity, and DNA damage [117]. Administration of 6′′′-p-coumaroylspinosin (P-CS) (flavonoid isolated from Ziziphi Spinosae Semen) on PC12 neuronal cells significantly prevents acrylamide-induced cell death, decreases GSH content, and ROS overproduction. P-CS was also suppressing the expression of Bax (apoptosis regulator) and Bim (pro-apoptotic protein) induced by acrylamide and inhibits the JNKs pathway [16].

Baicalein exerts protective effects in vivo and in vitro against 6-hydroxydopamine (6-OHDA) [118]. Baicalein prevented abnormal behavior by increasing dopaminergic neurons and dopamine and serotonin levels in the striatum and also inhibited oxidative stress and astroglia response [119]. Similarly, baicalein protects cells against the toxicity of a point mutation in α-synuclein [120], and inhibited the formation of α-synuclein oligomers, and consequently prevents its oligomerization [121]. Mitochondrial dysfunction in SH-SY5Y cells and upregulation of DJ-1 protein expression induced by 6-OHDA are prevented by baicalein [122]. Baicalein downregulates the activation of NF-κB, ERK, and JNK and attenuates astrocyte activation in MPTP mice [123].

Rutin protects dopaminergic neurons against 6-OHDA-induced neurotoxicity by and activating SOD, catalase, GPx, and total GSH activity and inhibition of LPO [124,125]. Kaempferol improves motor coordination, raises striatal dopamine and its metabolite levels, increases SOD and GSH activity, and reduces the content of LPO, also preventing the loss of TH-positive neurons induced by MPTP [126]. Kaempferol exhibit neuroprotection in models of rotenone-mediated acute toxicity by protecting SH-SY5Y cells and primary neurons from rotenone toxicity [127].

Quercetin protects against oxidative stress and increases activities of ATPase, SOD, GPx, Acetylcholinesterase, and dopamine depletion in MPTP-treated mice [128]. Furthermore, in a rotenone model, quercetin has been shown to upregulate mitochondrial complex-I activity and increase catalase and SOD activity [129]. In the 6-OHDA rat model, treatment of quercetin increased levels of antioxidants and striatal dopamine and reduced dopaminergic neuronal loss [130]. Luteolin also reduces cytotoxicity induced by 6-OHDA and ROS production in neuronal PC12 cells by modulating changes in the stress response pathway [131]. In MPTP-treated mice, luteolin and apigenin protect dopaminergic neurons by reducing oxidative damage, neuroinflammation, and microglial activation and also improve muscular and locomotor activity [92].

Baicalein prevented the progression of α-synuclein accumulation and protected dopaminergic neurons, and also inhibited the formation of α-synuclein oligomers in a rotenone mouse model [132]. Hesperidin (found in oranges and lemons) protects against iron-induced oxidative damage in the Drosophila melanogaster model of PD. Hesperidin restores dopamine levels, cholinergic activity, and improves motor function [133]. Antunes et al. found that hesperidin protects against neurotoxicity by reducing oxidative damage, increasing dopamine levels, and also improving the behavioral parameters in 6-OHDA-treated mice [134].

### 5.3. Role of Flavonoids in Proteotoxicity

Neurological disorders are marked by the presence of protein aggregates termed as amyloid, malfunctioned ubiquitin-proteasome system (UPS), and disrupted PQC network. These aggregates are present as insoluble prefibrillar amyloid-β oligomers (AβO) or insoluble amyloid-β oligomers [135,136,137,138]. In AD, the aggregated protein species, known as amyloid-β, are considered as the most neurotoxic species, while, in PD, the presence of α-synuclein aggregates and Lewy bodies are prominent hallmarks of PD pathology. These aggregates or disrupted UPS are the consecutive resultants of various stress conditions. Under stress conditions, the PQC system fails, thus being unable to combat proteotoxicity. Under such conditions, it has been found that flavonoids can effectively exclude proteotoxicity by preventing the formation of protein aggregates. Different cell model studies suggest that flavan-3-ols (especially their metabolites) could serve as great therapeutic targets for AD prevention. ‘Phenyl-γ-valerolactones (PVL)’ a flavan-3-ols’s metabolite efficiently reduces the Aβ-mediated toxicity. In yeast and mammalian cells, these PVLs especially monohydroxylated PVL, exclude the β-oligomer-induced toxicity and prevent cell death. Another PVL ‘(4′-OH)-PVL’ has been found to disrupt the Aβ assembly. Atomic force microscopy (AFM) images have shown the remodeling of toxic AβO aggregates into non-toxic amorphous aggregates [139]. Cellular protein aggregates hamper the PQC, thus causing disrupted protein homeostasis [140]. Myricetin (a type of flavone) inhibits aggregation of different aberrant proteins and modulates the HSP70 chaperone and quality control (QC)-E3 ubiquitin ligase E6-AP levels. Myricetin alleviates cytotoxicity by stabilizing the E6-AP, thus reducing the misfolded protein inclusions [141].

Modified flavonoids could be a promising candidate against various diseases. Dihydroquercetin, a modified form of quercetin, enhances the quercetin quality. Under physical stress conditions (thermal and chemical), quercetin fails to prevent stress-induced cell death. In contrast, dihydroquercetin has successfully prevented cellar injuries. Moreover, under hyperthermic stress, as well as sodium arsenite exposure to cells, quercetin led to a reduction in HSP70 synthesis and accumulation [142].

Pesticides cause various diseases as evidenced by many epidemiological studies. Mechanistic studies have shown their association with proteotoxicity as they induce the formation of Aβ amyloids. Silymarin, a flavonolignan extracted from the seeds of the milk thistle Silybummarianum, promotes the reduction of paraquat-induced Aβ aggregates formation in C.elegans [143]. Epimedium treatment on two C. elegans models of human proteotoxic disease namely CL4176 (expressing amyloid-β (1–42) peptide) and AM140 (expressing apolyglutamine protein), have shown the anti-proteotoxic property. Moreover, it also involves the reduction of Aβ1–42 and polyglutamine-induced paralysis in both models [144].

Treatment of 6′′′-feruloylspinosin (6-FS), one of the main active flavonoid components in Sour Jujube seeds, on the β-amyloid protein of transgenic C. elegans (GMC101) and PC12 cells resulted in delaying the aging process, reduced the rate of paralysis, enhanced the resistance to heat stress, increased the chemotaxis ability and promoted autophagy activity through the autophagy/lysosome pathway. Furthermore, 6-FS reduced the β-amyloid-induced toxicity by suppressing the deposition of β-amyloid and aggregation of the protein. It also increased the level of mitophagy in PC12 cells by promoting the expression of Pink1/Parkin in the mitophagy pathway [145].

### 5.4. Role of Flavonoids in Endoplasmic Reticulum (ER) Stress

ER stress is a condition caused by the accumulation of misfolded proteins and alterations in the calcium homeostasis which leads to the disruption of the structure and function of the ER. The ER stress response uplifts the expression of specific proteins including ER chaperones and proteins associated with the degradation of misfolded proteins. In ER stress, the accumulation of unfolded proteins disrupts the cellular proteostasis balance. This condition triggers the downstream signaling cascade in the ER, termed unfolded protein response (UPR). Prolonged ER stress induces several pathological conditions and aggravated ER stress may even lead to cell death. In several human neuronal pathologies, such as PD, AD, and Huntington’s disease (HD), ER stress has been reported. In recent years, the discovery of small molecules that could inhibit the UPR and ER stress have gained much attention to produce potential therapeutics [12,146,147,148,149,150].

Case studies on intake of diet rich in flavonoids have shown potential against many diseases. Kaempferol a natural flavonol attenuates the ER stress-induced cell death in human neuroblastoma cell line IMR32 via inhibiting the UPR signaling. Kaempferol significantly reduces the Brefeldin-A (BFA) induced mRNA expression of UPR markers like glucose-regulated protein (GRP78) and C/EBP homologous protein (CHOP) in IMR32 [146]. Luteolin, flavanol, is present in various plant products, such as celery and broccoli. Treatment with luteolin in PC12 cells has shown the attenuation of GRP78 and CHOP upregulation [131]. Apigenin treatment on murine HT22 hippocampal neuronal cells has shown a reduced level of ER stress-associated proteins including CHOP, GRP 78, and GRP94. Additionally, it has a role in the cleavage of activating transcription factor 6a, phosphorylation of eukaryotic initiation factor 2a, and inositol-requiring enzyme 1a, and the activation of mitogen-activated protein kinases, such as p38, c-Jun NH2-terminal kinase, and extracellular-regulated kinase [151]. Epicatechin (EC), a type of flavan-3-ol, has antibacterial, antitumor, antimutagenic, antiviral, and antioxidant properties. EC treatment on HT22 hippocampal neuronal cells successfully prevents the methamphetamine (METH) induced neurotoxicity. EC inhibits the activation of ERK, p38, CHOP, and DR4 expression [12]. Thus, ER-stress may be prevented using EC flavonoids. Effect of different flavonoids on the cellular stress response is summarized in Table 2.

## 6. Pre-Clinical/Clinical Studies of Flavonoids

After knowing the beneficial effects of flavonoids, several pre-clinical studies have been conducted to know the way of administration and the doses in animal models specific to AD, PD, Amyotrophic lateral sclerosis (ALS), and HD. A systematic review of the preclinical study on AD and PD suggested that flavonoids could be a potential drug to treat neurodegenerative diseases [157]. The possible mode of action, dose, and route of administration are summarized in Table 3.

An epidemiological study on 808 adults Italian cohort found that higher dietary intake of anthocyanins, flavan-3-ols, catechins, and flavonols are associated with better cognitive health [158]. Intake of dietary flavonoids can mitigate the pathogenesis of neurological disorders by reducing oxidative stress. A cohort study performed on 1367 elderly (more than 65 years) depicted that flavonoid intake is inversely related to the risk of incident dementia [159].

## 7. Flavonoid Metabolism

To use flavonoids as a therapeutic agent, it is important to know their pharmacokinetics. As these dietary flavonoids are used as traditional medicines from past decades, many studies have been conducted to know their absorption and metabolism to rule out their possible way of action. Dietary flavonoids are mostly found in the glycoside form. After ingestion of these dietary flavonoids, the deglycosylation process occurs in the small and large intestine. Lactase-phlorizin hydrolase (LPH) is the first enzyme reported for the hydrolysis of quercetin 3-O-glucoside (Q3G) and quercetin 4′-O-glucoside (Q4′G) that are monoglucosides of genistein and daidzein to produce aglycons in-vitro [174]. Before hydrolysis, the glucosides are taken up into the cells via sodium-glucose co-transporter type 1 (SGLT1) membrane transporter and this is reported for Q4ʹG, which was found using human Caco-2 cells and SGLT1 transfected rodent G6D3 cells [175]. After the hydrolysis, the produced aglycons are inserted in the epithelial cells and metabolized via phase II enzymes to produce corresponding conjugated metabolites. These phase II enzymes are uridine-5ʹ-diphosphate-glucuronosyltransferases (UGT), sulfotransferases (SULT), and catechol-O-methyltransferases (COMT) [176]. After intestinal conjugation, further conjugation including sulfation and methylation occurs in the liver. Post metabolism, several chemical forms of flavonoids are found in the urine, and systemic circulation [177,178,179]. After excretion, metabolites are further deconjugated by the microbiota and reabsorbed. The transportation of these absorbed flavonoids is conducted via the lymph in the body [180]. Flavonoids are found in the form of conjugated metabolites in the blood and tissues which are reported to have lower activity than the aglycon form. The functions of flavonoid metabolites are controlled by the balance of the conjugation-deconjugation process.

## 8. Neuronal Access of Flavonoids

There exists a lacuna of experimental evidence of whether flavonoids can cross the BBB [181]. This lacuna hinders the development of flavonoids-based therapeutics. Daily intake of dietary flavonoids is beneficial for many neurodegenerative disorders as supported by epidemiological studies. Therefore, numerous studies are being conducted to enhance access and promote the neuronal accessibility of flavonoids.

A study on the human brain endothelial cell line (HBMEC) model has revealed that amongst three flavonoids: quercetin, epigallocatechin gallate (EGCG), and cyanidin-3-glucoside (C3G), EGCG crosses the BBB more rapidly than C3G while quercetin was unable to cross BBB. Another study conducted on eighteen, three-month-old male Sprague-Dawley rats showed that quercetin can cross the BBB if administered with α-tocopherol (Vitamin E) [182]. These studies have proven that flavonoids can be used in combating neuronal disorders since they can reach the site of damage (by crossing the BBB) and exert their therapeutic effect.

## 9. Flavonoid Extraction: A Key to Improved Flavonoid Property

The traditional method for flavonoid extraction reduces its quality. Thus, recent studies have gained attention for improving the flavonoid property by modulating the extraction method. Many approaches, such as solvent extraction (SE), microwave-assisted extraction (MAE), supercritical fluid extraction (SFE), ultrasound-assisted extraction (UAE), and ultra-high pressure extraction (UHPE), are gradually being used for improving the content and quality of flavonoid [183].

**Figure 1:**
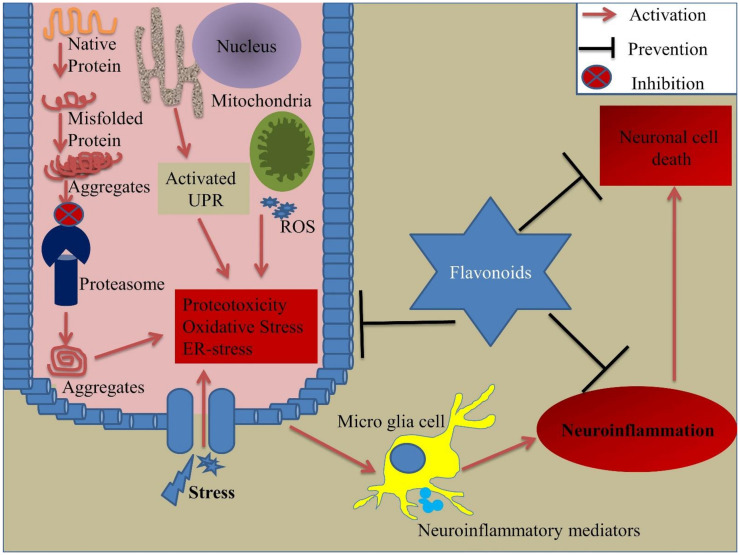
Role of flavonoids in prevention against cellular stress response. Exposure to stress conditions leads to the activation of cellular stress responses, such as UPR, ER stress, oxidative stress, proteotoxicity, and neuroinflammation. When cells are exposed to any stress condition, it affects the cellular proteome, thus inducing the UPR in the ER and further activation of ER stress. Stress conditions also initiate proteotoxicity by affecting the proteins’ structure, as well as proteasome subunits of the proteasomal degradation machinery, causing the release of misfolded/aggregated proteins in the cytosol, thus inducing proteotoxicity. The release of ROS from mitochondria leads to the generation of oxidative stress. All these cellular stress responses try to eliminate the stress-induced toxicity, but extreme cellular stress responses may lead to cell death. During stress exposure, microglia start to release neuroinflammatory mediators thus causing neuroinflammation. This inflammation creates a hostile environment within the cell and under harsh conditions, leads to cell death. Flavonoids have the potential to combat and prevent these exaggerated cellular stress responses in-turn preventing cell death. ER: Endoplasmic reticulum, ROS: Reactive oxygen species, UPR: Unfolded protein response.

The fruits of Ziziphus jujube Mill., known as jujube or Chinese date have neuroprotective properties. Jujube protects neuronal cells against neurotoxin stress, promoting memory and learning, stimulating neuronal differentiation, and increasing the expression of neurotrophic factors [183]. Flavonoids extracted from jujube seed by using the UAE method improves its medicinal quality [184,185]. Moreover, jujube seed flavonoid extracted by UAE method displayed a higher capacity of scavenging ABTS, DPPH, superoxide, and hydroxyl radicals and reducing the level of ROS accumulation in PC12 cells. Moreover, administration of these flavonoids in the transgenic *C. elegans* model (GMC101) reduces the Aβ toxicity [17]. The UHPE method has many advantages, such as shortening the time, reducing the temperature, and reducing the solvent. Flavonoid extracted from jujube seed through UHPE shows higher concentrations of total flavonoids extracted and stronger DPPH and ABTS radical-scavenging activities in a shorter period [186]. Thus, applying the improved flavonoid extraction method would be beneficial for improving the flavonoid property. The role of flavonoids in prevention against oxidative stress, neuroinflammation, and ER stress is summarized in Figure 1.

## 10. Conclusions

Current data on neurodegenerative disorders suggest the need for a potential therapeutic target. With a deep understanding of the neurological pathologies, it becomes easy to target the potential hallmarks that are responsible for these diseases. Flavonoids are phytochemicals, and many studies on these compounds depict their effective role against neurological disorders. Flavonoids have shown beneficial effects on the cellular stress response. As described by several studies, these flavonoids could be promising candidates for neurological disorders. Further studies are needed to focus on their clinical acceptance. Modified flavonoids also need to be studied in detail to assess their role as therapeutics in neurological disorders. Risk assessment and pharmacokinetics of flavonoids are essential parameters that need to be explored for their clinical use. Hence, a multi-fold increase in the number of in-vivo and clinical studies is the need of the hour.

## Figures and Tables

**Table 1:** A subgroup of flavonoids, their natural resources, and example.

Subgroup	Chemical Structure	Plant Source	Example	Ref.
Isoflavones	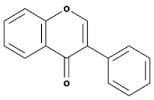	Soybeans, leguminous plants, microbes,	Genistein, Daidzein, Glycerin, Formanantine	[71,72,73,74]
Flavones	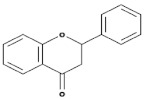	Leaves, flowers, and fruits	Luteolin, Apigenin	[75,76]
Flavanones	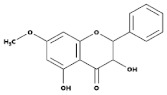	All citrus fruits	Hesperidin, Naringenin	[77]
Flavonols	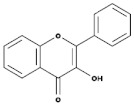	Onions, berries, lettuce, tomatoes, grapes, and apples	Kaempferol, Quercetin	[78]
Neoflavonoids	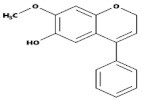	Sri Lankan endemic plant *Mesuathwaitesii*	Calophyllolide	[79,80]
Flavanols(Flavan-3-ols)	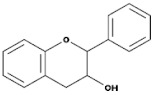	Peaches, pears, blueberries, bananas, and apples	Catechins, Epicatechins, Epigallocatechin	[81,82]
Anthocyanins	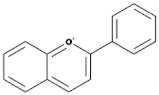	Bilberries, cranberries, merlot grapes, blackberries, black currants, red grapes, strawberries, blueberries, and raspberries	Cyanidin, Delphinidin, Malvidine	[83]
Flavones	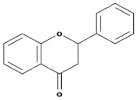	Leaves, flowers, and fruits	Luteolin, Apigenin	[84,85]
Flavanones	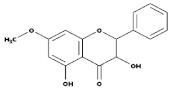	All citrus fruits	Hesperidin, Naringenin	[77]

**Table 2:** Effect of flavonoids on the cellular stress response.

Flavonoids	Cellular Stress Response	Host Model	Ref
Kaempferol	Inhibits the expression of GRP78 (a chaperone) and *CHOP* (ER stress associated pro-apoptotic transcription factor)	Human IMR32	[146]
Quercetin	Reduction in the expression of glucose-regulated protein 78 (GRP78) and C/EBP-homologous protein (CHOP)	Human umbilical vein endothelial cells	[152]
Morin	Inhibition of the expression of GRP78, Decreased ROS and apoptosis	renal proximal tubular HK-2 cells	[153]
Methoxyflavones	Activation of the UPR pathway via activating eIF2α and Nrf2 and induces the expression of downstream genes, such as GRP78, HO-1, and CHOP, without causing ER stress	Mouse insulinoma MIN6 cells	[147]
Agathisflavone	Increases the remyelination and alters microglial activation state. Neuroprotective effect via increase the expression of neurotrophic factors ciliary neurotrophic factor (Cntf), epidermal growth factor receptor (Egfr), and neuronal GABA b1 receptor subunit (Gabrb1)	Mice belonging to the C57BL/6 background	[154]
Apigenin	Neuroprotection, astrocytes integrity and have an anti-neuro-inflammatory response. These responses are generated via the modulation of inflammatory cytokines mRNA expression and reduce the expression of OX42, IL-6, and gp130. Induces the expression of brain-derived neurotrophic factor (BDNF).	Wistar rats’ hemispheres brain’s Glial cells and neurons	[154]
Hesperetin	Reduction of the expression of inflammatory Cytokines by ameliorating Toll-like receptor-4 (TLR4)-mediated ionized calcium-binding adapter molecule 1/glial fibrillary acidic protein (Iba-1/GFAP) expression. Attenuation in the LPS-induced generation of reactive oxygen species/lipid peroxidation (ROS/LPO) and improved the antioxidant protein level, such as nuclear factor erythroid 2-related factor 2 (Nrf2) and Haem-oxygenase (HO-1), in the mouse brain	C57BL/6 N mice	[155]
Epimedium	Have anti-proteotoxic potency as it reduces the Aβ1–42- and polyQ-induced paralysis in CL4176 and AM140	*C. elegans* human proteotoxic disease models (CL4176, AM140)	[144]
Rutin	Rutin treatment reduces polyglutamine (polyQ) protein aggregation in muscle, reduced polyQ-mediated neuronal death in ASH sensory neurons, and extended lifespan.	*C. elegans* model of Huntington’s disease	[156]
phenyl-γ-valerolactones (metabolites of flavan-3-ols)	(4′-OH)-PVL interferes with AβO (but not fibril) assembly and actively remodels performed AβOs into nontoxic amorphous aggregate.	Yeast strains expressing different variants of the human A*β*42 and *β*23 peptides	[139]

**Table 3:** Studies related to the effect of flavonoids on the animal model.

Disease	Clinical Onsets	Behavioral Onsets	Disease Model	Flavonoids	Dose	Effect of Flavonoids Treatment on the Animal Model	Ref.
Alzheimer’s disease (AD)	Presence of extracellular neuritic plaques containing (Aβ) peptide and intracellular neurofibrillary tangles containing tau	AD results in a progressive loss of cognitive ability and eventually daily function activities	5 × FAD model	7,8-dihydroxyflavone (7,8-DHF)	IP injection (5 mg/kg)	Improved memory	[160]
					Oral administration (5 mg/kg/day)	Improvement in memory and reduction in synapse loss	[161]
			2 × FAD model	Apigenin	Oral administration (40 mg/kg/day)	Improvement in learning and memory, reduction in deposition of insoluble Aβ	[162]
			1 × FAD model, 3 × FAD model, SAMP8 mice	Nobiletin	IP injection (10 mg/kg)	Improvement in memory and reduction in levels of both soluble and insoluble Aβ	[163]
					IP injections (10 and 30 mg/kg)	Improvement in memory; reduction in soluble Aβ levels	[164]
			1 × FAD model	Baicalein	IP injections 10 and 50 mg/kg	Improves the memory, reduces some markers of oxidative stress	[162]
			(SAMP8)	Quercetin	IP injections (10 mg/kg)	Improves working memory and reduces the production of Aβ	[165]
					Oral administration (25 mg/kg/day)	Reduces the markers of oxidative stress, LPO and activates the ERK pathway	
Huntington’s disease (HD)	Presence of a trinucleotide repeat (CAG) that encodes an abnormally long polyglutamine tract in the huntingtin protein	Movement and psychiatric disturbances, as well as cognitive impairment	3-NP model of HD in rats	Chrysin	Oral administration (50 mg/kg/day)	Improvement in behavior and reduction in markers of oxidative stress and cell death, and enhancement in the survival of striatal neurons	[166]
			R6/1 N-terminal transgenic mouse model	7,8-DHF	Oral administration (5 mg/kg/day)	Delay the development of motor and cognitive deficits, prevention of the loss of striatal volume, enhances the marker of neurotrophic factor signaling, and reduction in some markers of inflammation	[167]
			3-NP model	Quercetin	oral administration (25 mg/kg/day)	Reduce motor deficits, improve mitochondrial function, and attenuate some markers of oxidative stress	[168]
			R6/1 N-terminal transgenic mouse model	Anthocyanins	100 mg/kg/day	Delay the loss of motor function	[169]
			3-NP model in rats	Hesperidin	Oral administration (100 mg/kg/day)	Reduce motor deficits, as well as markers of inflammation and oxidative stress	[170]
Amyotrophic Lateral Sclerosis (ALS)	Heritable gene mutations	Loss of the motor neurons that control the voluntary movement of muscles, resulting in paralysis and death	SOD1-G93A model	7,8-DHF	IP injection (5 mg/kg)	Reduction in the age-dependent decrease in motor performance and preserving the total motor neuron count and dendritic spine density on motor neurons	[171]
				Fisetin	Oral administration (9 mg/kg)	Delay the development of motor deficits, reduction in their rate of progression, and increases lifespan	[172]
				(−)-epigallocatechin gallate (EGCG)	oral administration (5.8–10 mg/kg)	Delay symptom onset and extend the lifespan	[173]

## Data Availability

Not applicable.

## Abbreviations

AD	Alzheimer’s disease
PD	Parkinson’s disease
HD	Huntington’s disease
ALS	Amyotrophic lateral sclerosis
ER	Endoplasmic reticulum
PQC	Protein quality control
NO	Nitric oxide
ROS	Reactive oxygen species
UPR	Unfolded protein response
HSP	Heat shock protein
